# Development and Evaluation of a Screening Tool to Aid the Diagnosis of Cluster Headache

**DOI:** 10.3390/brainsci10020077

**Published:** 2020-02-01

**Authors:** Alina Buture, Jason W Boland, Lisa Dikomitis, Chao Huang, Fayyaz Ahmed

**Affiliations:** 1 Hull York Medical School, University of Hull, Hull HU6 7RX, UK; jason.boland@hyms.ac.uk (J.W.B.); chao.huang@hyms.ac.uk (C.H.); fayyaz.ahmed@hey.nhs.uk (F.A.); 2 School of Medicine, Keele University, Newcastle-under-Lyme ST5 5BG, UK; l.a.dikomitis@keele.ac.uk; 3 School of Primary, Community and Social Care, Keele University, Newcastle-under-Lyme ST5 5BG, UK; 4 Department of Neurology, Hull University Teaching Hospitals NHS Trust, Hull HU3 2JZ, UK

**Keywords:** migraine, images, drawings, questionnaire, sensitivity, specificity, survey, intervention development

## Abstract

Cluster headache (CH), a severe primary headache, is often misdiagnosed and mismanaged. The aim of this study was to develop and evaluate a screening tool to aid the diagnosis of CH. We developed a novel 12-item screening tool. This was comprised of four components: (1) images depicting headache pain; (2) pain descriptors; (3) key questions that could differentiate between CH and migraine; and (4) a visual analogue pain scale. The total possible questionnaire score ranged from 3-32. Patients with CH and migraines (control group) were recruited prospectively from a headache centre in the North of England, UK. Two-hundred and ninety-six patients were included in the study: 81 CH patients, 36 of which suffer with episodic CH and 45 with chronic CH; 215 migraine patients, 92 of which suffer with episodic migraine and 123 with chronic migraine. The mean questionnaire score was higher in CH patients versus migraine patients (28.4 versus 19.5). At a cut-off score of >25 out of 32, the screening tool had a sensitivity of 86.4% and a specificity of 92.0% in differentiating between CH and migraine. The screening tool could be a useful instrument to aid the diagnosis of a CH. The images depicting headache pain do not clearly discriminate between CH and migraine.

## 1. Introduction

A cluster headache (CH) is a severe primary headache with a prevalence of approximately 0.1% [1]. CH patients incur a high healthcare cost, estimated in the USA as greater than $2.8 billion/year [2]. CH is characterised by trigeminal distribution of pain, cranial autonomic symptoms and circadian and circannual periodicity [3]. Research indicates that the most common misdiagnosis of CH is migraine [4,5,6,7,8]. Although CH has very distinct clinical features, patients often face delay in diagnosis, misdiagnosis, and mismanagement [6,8,9,10,11]. It is important that CH is diagnosed early as effective therapies exist and should be recommended [12]. Misdiagnosis could be avoided if healthcare professionals (such as primary care practitioners, clinicians in secondary and emergency care) are aware of the striking differences between the clinical presentation of CH and that of migraine. Despite the significant disability and impact on quality of life associated with CH, patients are often in a diagnostic limbo for many years, living with debilitating, severely painful attacks, before a correct diagnosis is made [13]. Misdiagnosis of CH has a significant impact on patients’ daily life, employment, and mental health [14]. Sick leave is high among CH patients [15]. A correct and timely diagnosis will improve the quality of life, will avoid unnecessary consultations and referrals, and, as a consequence, will reduce the financial and human health resource burden on the healthcare system [13]. Even though the diagnostic delays of CH have decreased over the past decades [16], the timeframe between the onset of the disease and first consultation at a headache centre is still high [9].

Despite significant advances in our understanding of CH pathophysiology [17,18,19] and treatment [20,21], the way CH is diagnosed remains unchanged [3]. To date, since there are no available biological markers to diagnose CH [3], the diagnosis is entirely based on the clinical history. The lack of knowledge of CH’s clinical characteristics could lead to incomplete history taking and the misdiagnosis of CH. There is a need for a screening tool to aid healthcare professionals to recognise CH. A pilot study by our research team, for which we developed a screening tool with six images depicting headache pain, demonstrated the potential of using visual aids to detect headache severity [22]. Here, we report the results of our large study in which we tested a screening tool for CH.

The aim of this study was to determine the overall performance of a screening tool and the performance of each item in the tool in differentiating between CH and migraine. We also aimed to determine the performance of images depicting headache pain in discriminating between CH and migraine.

## 2. Materials and Methods

### 2.1. Study Design

This is a prospective case-control study, evaluating a newly developed 12-item self-administered questionnaire. The study included patients with CH (the study group) and a group of patients with migraines (control group).

### 2.2. Study Population

All patients were recruited prospectively from a headache centre in the north of England, UK, between October 2017 and March 2019. Patients older than 18 who received a prior diagnosis of CH or migraine based on the ICHD-3b criteria [23] were invited to participate by A.B. and F.A. Patients with a dual diagnosis of CH and migraine were excluded from the study.

### 2.3. Ethics

This study has received ethical approvals from the local University Research Ethics Committee (reference no: 1613/27.09.2016) and from the Health and Social Care Research Ethics Committee (HSC REC) (reference no: 16/NI/0269). All patients provided written informed consent to participate. The patients completed the questionnaire un-aided.

### 2.4. Screening Tool Development

**Figure 1:**
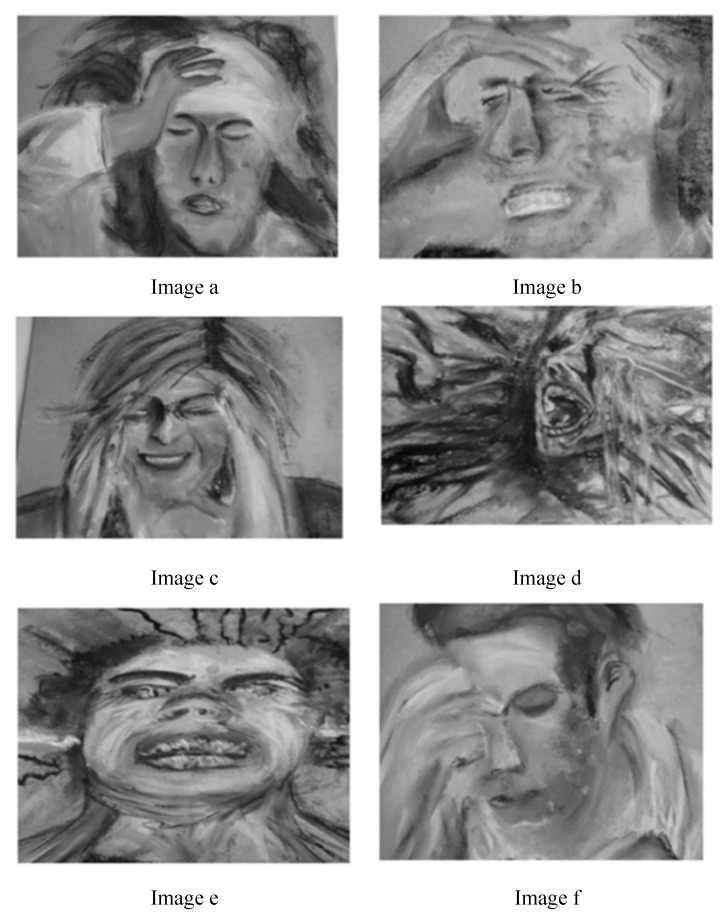
Images depicting different pain severities.

The screening tool comprised of four main components: (1) screening tool with six images depicting headache pain (Figure 1) [22]; (2) verbal description of pain; (3) key questions that could differentiate between CH and migraine; and (4) a visual analogue scale. The images used in the first component were inspired on real life pictures and artistic renditions of headache available online [24,25,26,27]. Image ‘a’ was inspired on a piece of artwork by Agnes-Cecile for Arte Cluster [22,24,25], and Image ‘e’ was inspired on art created by Faderhead for Deviant Art (Figure 1) [27]. The methodology on how we developed this screening tool with six images depicting headache pain severity has been published [22]. Healthy participants rated image ‘d’ and image ‘e’ as excruciating, image ‘b’ as severe, image ‘c’ as severe/moderate, and image ‘f’ as depicting mild pain [22]. The International Classification of Headache Disorders-3b (ICHD-3b) [23] criteria and the patients’ description of pain in the Cluster Headache: Impact and Perception Study (CHIPS) [28] were used to determine the verbal description of pain, which included categories such as the intensity of the pain (mild, moderate, severe, very severe, excruciating), the nature of the pain (pressure, throbbing, stabbing, burning), and a description of the pain (red hot poker in the eye, pounding heart in the head) [29]. The key questions were provided by 10 UK-based headache specialists, members of the British Association for the Study of Headache (BASH) [30,31]. The headache experts were invited to participate via email. They were asked to provide questions that they thought were able to differentiate between CH and migraine during a clinical consultation. The most asked questions provided by the headache specialists were included in the screening tool.

The questionnaire was pilot tested on patients with CH and migraine. Concerns were raised by the patients with migraine as to whether they should report their mild headaches or migraine attacks. To avoid confusion, the questionnaire was customised based on diagnosis (i.e., ‘Please choose one image that best illustrates your cluster headache attacks?’ versus ‘Please choose one image that best illustrates your migraines?’) (see Supplementary File).

### 2.5. Statistical Methodology

#### Analysis

The dataset was analysed via descriptive analysis [32]. The frequency distribution (counts and percentages) of the categories within each of the 12 variables used to evaluate the performance of the screening tool were summarised for each group of patients. The test scores were coded numerically as defined in Table 1. Higher scores were given for test items characteristic for CH. A dichotomous scale (no = 0; yes = 1) was used for the test items with binary responses (restlessness, excruciating agony, headache at specific times, strictly unilateral pain, ipsilateral cranial autonomic symptoms). Test items for the severity of pain were coded as follows: image preference (f = 1 (least severe); a = 2; c = 3; b = 4; e = 5; d = 6 (most severe)) [22], the pain scale (scores from zero to ten), and the intensity of pain (mild = 1; moderate = 2; severe = 3; very severe = 4; excruciating = 5). The description of pain as a ‘red hot poker in the eye’, which is usually attributed to CH [29], was coded with 1, whilst a ‘pounding heart in the head/other’ was coded with 0. A ‘stabbing/burning’ pain that describes CH [33] was coded with 3, while ‘pressure’ was coded with 2, and ‘throbbing/other’ with 1. The attack duration of ≤ 3 h, which characterises CH, was coded with 1, whilst the attack duration > 3 h was coded with 0. A total score was determined by adding up the scores for the 12 items in the screening tool [34]. The total score was analysed to evaluate the overall performance of the screening tool. Table 2 shows that the minimum possible total score = 3, and the maximum possible total score = 32. The descriptive statistics (mean ± 95% CI) of the total score were compared between each group of patients.

### 2.6. Sensitivity and Specificity and the Receiver Operating Characteristics (ROC)

Specificity, sensitivity, false positive rate, false negative rate, positive and negative predictive values, and positive and negative likelihood ratios were calculated for the items with dichotomous responses [35]. Descriptive statistics (mean and 95% confidence intervals) and ROC curve statistics were computed to determine how the total scores could be interpreted to distinguish between patients diagnosed with CH versus migraine [36]. The ROC curve also permitted the identification of a cut-off test score that best distinguished between patients with CH and migraine. This cut-off test score was indicated by the inflection point on the ROC curve that was closest to the top left corner [37]. We also performed gender segregated analysis as follows: we separated the data set into male and females. For females, we performed class balancing to equalise the number of occurrences of CH and migraine. For males, we did not perform balancing as the data set is approximately balanced. A total sample size of about 300 subjects is generally required to provide accurate estimates for sensitivity and specificity for most screening or diagnostic tests involving two groups of patients, among which a specified disease is either present or absent [38]. The analysis was performed using the software R version 3.5.1.

## 3. Results

### 3.1. Description of the Sample

The sample consisted of 296 patients, which were classified into the following two groups: the case group and the control group. The case group consisted of patients diagnosed with CH (*n* = 81, 27.4%), of whom 45 patients were diagnosed with chronic CH (55.6%) and 36 with episodic CH (44.4%). The control group consisted of patients with migraine (*n* = 215, 72.6%), of whom 123 were patients with chronic migraine (57.2%) and 92 patients with episodic migraine (42.8%). The ages of the patients ranged from 18 to 79 years (mean = 43.8; 95% CI = 42.3, 45.6). Patients with CH had a mean age of 46.06 (95% CI = 43.18, 44.94) and patients with migraine had a mean age of 42.93 (95% CI = 41.02; 44.83). The CH group was comprised of 51 males and 30 females (male:female ratio: 1:7) whilst the migraine group consisted of 35 males and 180 females (female:male ratio: 5:14). The females had a mean age of 42.8 (95% CI 41; 44.7) whilst males had a mean age of 46 (95% CI 42.9; 49.1).

### 3.2. Descriptive Analysis of Test Scores

Table 3 summarizes the frequency distributions of the test scores classified by diagnosis. The image preference with the highest frequency was image ‘d’ for CH (61.9%); whereas image ‘b’ (21.5%) and image ‘c’ (23.3%) were the highest frequencies for migraine. Image ‘d’ was rated as showing excruciating pain by most healthy participants in a study by our research team, while image ‘c’ was rated as moderate/severe and image ‘b’ as severe [22].

The description of pain with the highest frequency was ‘red hot poker in the eye’ for patients diagnosed with CH (69.2 %), whereas ‘pounding heart in head/other’ was the highest frequency for patients diagnosed with migraine (84.4%). Most patients diagnosed with CH (90%) reported the presence of restlessness during the attacks, compared to less than half of the patients with migraine (43.2%).

Most patients diagnosed with CH (96.4%) reported the presence of ‘ipsilateral cranial autonomic symptoms’, whereas most patients diagnosed with migraine (57.8%) reported the absence of ‘ipsilateral cranial autonomic symptoms’.

### 3.3. Sensitivity and Specificity of the Test Scores

Table 4 shows the sensitivity and specificity statistics for the eight test items that used dichotomous responses.

The presence of ‘ipsilateral cranial autonomic symptoms’ had a high sensitivity (96%) but a moderate false positive rate (43%). ‘Excruciating agony’ also had a high sensitivity (93%) but a higher false positive rate (67%). ‘Restlessness’ was a symptom with high sensitivity (90%) and a moderate false positive rate (44%). ‘Strictly unilateral attacks’ had a high specificity (86%) and a moderate false positive rate (44%). The ‘Description of pain’ had a lower level of sensitivity (68%) than the other symptoms, but it also had a lower false positive rate (16%). ‘Headaches at specific times’ had a low sensitivity (60%) but also with a low false positive rate (19%).

### 3.4. ROC Analysis for Whole Data Set

**Figure 2:**
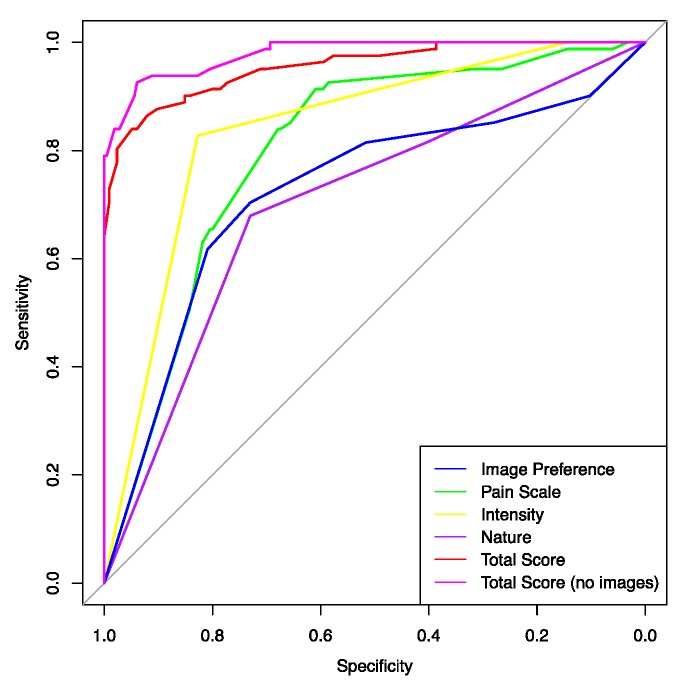
Receiver Operating Characteristics (ROC) curves for whole data set. The area under the curve (AUC) Total score = 0.955; Total score (no images) = 0.979; AUC Intensity = 0.841; AUC Pain scale = 0.799; AUC Image preference = 0.723; AUC Nature of pain = 0.702; AUC; area under the ROC curve is significantly greater than 0.5 (*p* < 0.001).

Figure 2 illustrates the ROC curves for ‘Image preference’, ‘Pain scale’, ‘Intensity of pain’, ‘Nature of pain’, and ‘Total score’. The areas under all of the ROC curves were >0.5 (*p* < 0.001), implying that the specified variables significantly distinguished between patients with CH and patients with migraine. None of the tests were worthless. The ROC curves also permitted the identification of cut-off test scores that best distinguished between patients with CH and patients with migraine. The cut-off scores are identified in Table 5. Based on the area under the curve (AUC), the most accurate test was ‘Total score’ (0.955 = good); followed in order of magnitude by ‘Intensity of pain’ (0.841 = good); ‘Pain scale’ (0.799 = fair); ‘Image preference’ (0.723 = fair); and ‘Nature of pain’ (0.702 = fair). The ‘Total score’ for the 12 items appeared to provide a more accurate method to distinguish between patients with CH and patients with migraine than the separate scores for ‘Image Preference’, ‘Pain scale’, ‘Intensity of pain’, and ‘Nature of pain’. When the images are removed, the ‘Total score’ is a more accurate test (sensitivity 92.6%, specificity 93.9%) than the ‘Total score’ of the 12 items (sensitivity 86.4%, specificity 92.0%). Patients with CH had a higher mean score (28.4; 95% CI 27.7; 29, 1) compared to patients with migraine (19.5; 95% CI 19; 20).

### 3.5. ROC Analysis According to Gender

**Figure 3:**
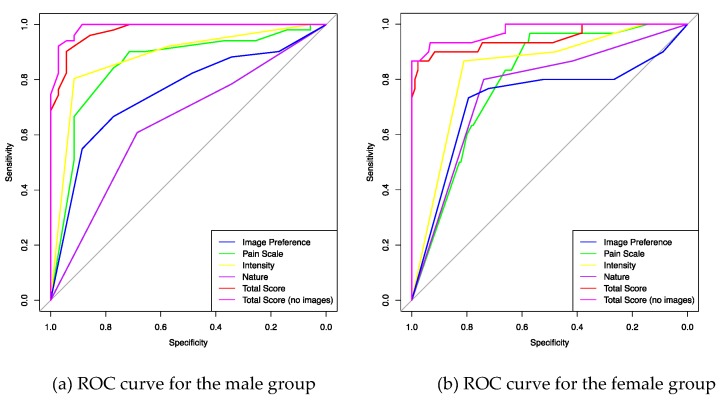
ROC curves according to gender. The AUC male group: AUC Total score = 0.977; AUC Total score (no images) = 0.979; AUC Intensity = 0.881; AUC Pain scale = 0.852; AUC Image preference = 0.751; AUC Nature of pain = 0.640.

Figure 3 shows the ROC curves according to gender. The cut-off points on the ROC curves are presented in Table 6. Similar to the analysis including the whole data set, the ‘Total score’ is the most precise test in differentiating CH from migraine. The ‘Total score’ >23 (out of 32) has a high performance (sensitivity 90.1%, specificity 94.2%) in detecting males with CH. The ‘Total score’ >25/32 has a lower sensitivity in detecting females with CH (sensitivity 90.0%, specificity 91.6%). Without the images, the ‘Total score’ has a slightly better performance than the ‘Total score’ of the 12-items for both male and female groups. The intensity of pain ‘excruciating’ has a higher specificity in detecting males with CH (91.4%) than females (81.1%). The image preference has a higher performance in detecting females (sensitivity 73.3%, specificity 79.4%) than males with CH (sensitivity 66.6%, specificity 77.1%).

The AUC female group: AUC Total score = 0.948; AUC Total score (no images) = 0.975; AUC Intensity = 0.838; AUC Pain scale = 0.795; AUC Image preference = 0.726; AUC Nature of pain = 0.761. The area under the ROC curve is significantly greater than 0.5 (*p* < 0.001).

### 3.6. ROC Analysis with Class Balancing

**Figure 4:**
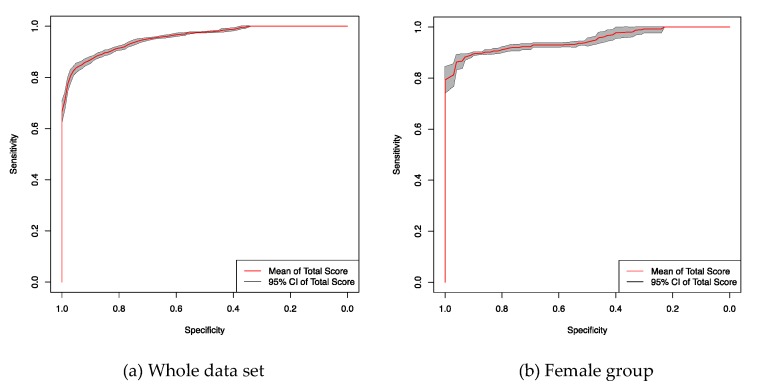
ROC curve of the ‘Total score’ after class balancing.

To evaluate the influence of the unbalanced classes on the statistical analysis in Figure 2 and Figure 3 we performed ROC analysis with class balancing. The data set is unbalanced in two ways: more females than males and more occurrences of migraines than CH are in the data set. Figure 4 shows the mean ROC curve and associated 95% CI of the ‘Total score’ for the whole data set and for the female group, respectively, after class balancing. Balancing was performed by gathering 10 random under-samplings of the occurrences of migraine from the complete data set, including both males and females. The ROC curve for each realization of the random under-sampling were averaged to obtain the presented ROC curves. Class balancing does not alter the characteristics of the ROC curve.

## 4. Discussion

In this study, we developed a self-administered screening questionnaire for CH. The main objectives of this study were to determine the overall performance of the questionnaire and the performance of the test items that best discriminate between CH and migraine. The results were interpreted assuming that a screening test should ideally exhibit a high level of sensitivity (to detect as many true positives as possible).

### 4.1. Clinical Indicators of CH versus Migraine

Current research indicates that the key differences between CH and migraine are the severity of pain, restlessness behaviour during attacks [3], and prominent ipsilateral cranial autonomic symptoms [39]. CH is often described in the literature as the ‘most severe pain known to man’ [28,40,41,42]. Such a pain descriptor is not usually attributed to migraines [43]. The attacks described like an ‘excruciating agony’ in our study proved to have a low specificity (33%). Although ‘excruciating agony’ had a high sensitivity (93%) in detecting CH, almost half of the patients with migraine (43%) chose this descriptor of pain. Therefore, the descriptor ‘excruciating’ might not apply only to CH patients [40] but also describes the severity of migraine attacks, as shown by our study. The descriptor of pain ‘excruciating agony’ is not a good discriminator between CH and migraine, according to our questionnaire study. When patients had to choose the intensity of pain from mild to excruciating, this descriptor of pain was a more accurate test (sensitivity 82.7%, specificity 82.7%). This highlights the importance of question phrasing that could influence the pain information provided [44]. Furthermore, pain reporting is subjected to multiple biases. In a stressful situation, recall of pain intensity is exaggerated and also chronic pain is itself associated with an overestimation of pain intensity [45]. Additionally, associated psychiatric co-morbidities could have an influence on how pain is reported [46]. The self-report of physical pain can be subjected to recollection bias [45,47]. Patients with CH overestimated the severity of retrospective attacks compared to the attacks recorded prospectively [48].

It is known from the literature that CH has very characteristic clinical features: restlessness during attacks and ipsilateral autonomic cranial symptoms [3]. These clinical characteristics had a high sensitivity (>90%) but with low specificity (<60%) which meant they were present in nearly all patients with CH and also present in many patients with migraine. Previous studies report variable results for the presence of restlessness during CH attacks (80% [49], 67.9% [10], 51% [50]). This could be due to differences in question phrasing even though the questionnaires used for data collection were not published [10,49,50]. According to previous reports, the presence of restlessness had a higher sensitivity in patients with chronic CH (sensitivity 100%, specificity 90%) than episodic CH (sensitivity 82%, specificity 92%) [51]. Although the cranial autonomic symptoms have been reported in 56% of patients with migraine, they are less severe, usually bilateral and inconsistent from one attack to the other [39]. In contrast, the cranial autonomic symptoms reported in 94% of patients with CH are severe, unilateral, and consistently present from one attack to the other [39]. In our study, the presence of ipsilateral cranial autonomic symptoms were reported in 96.4% of patients with CH and 42.2% of patients with migraine, similar to previous reports [39].

More research is required to identify the descriptors of pain that best differentiate between CH and migraine. Untreated attack duration (3 h) was the test item with the highest sensitivity (100%) and specificity (100%). Previous reports showed similar findings [51,52,53]. According to previous studies, the untreated attack duration of ≤3 h is one of the best clinical features to discriminate between CH and other primary headaches [51,52,53]. However, this is the result of strictly applying the ICHD-3b criteria [3], where CH patients with attacks longer than 3 h are excluded from the research studies. This study showed many overlapping features between CH and migraine which could account for diagnostic delays and misdiagnosis. There is no single test item that can differentiate between the two conditions. However, the total screening tool score with a cut-off > 25/32 was highly sensitive (86.4%) and specific (92.0%) in differentiating CH from migraine. This tool is designed as a screening instrument, and further confirmation of diagnosis from a specialist is required.

### 4.2. Screening Tools for CH

Multiple proposals have been made for screening and diagnostic tools for CH [34,52]. A screening tool with 8-items was found to have a high sensitivity (95%) and specificity (96%) in detecting CH [34]. This study, which included 42 patients with CH, of whom one patient had chronic CH, should be repeated in a higher population of patients with chronic CH to avoid selection bias [34]. A lengthy 142-item web based questionnaire was tested in a large population of 437 patients with self-reported CH [52]. Three questions were identified to predict CH: untreated attack duration of 15 to 180 min, an attack free period of four months to three years, and the male gender (sensitivity 53.8%, specificity 88.9%, positive predictive value 95.5%, and negative predictive value 30.8% [52]). The three-item questionnaire was not independently tested and the study did not include a control group [52]. Fritsche et al. tested a 20-item German language questionnaire to diagnose migraine, tension type headache, and trigeminal autonomic cephalalgias (TACs) in a tertiary headache centre [54]. The exact types of TACs are not specified and therefore the number of diagnosed CH cases is unknown [54]. The same questionnaire developed by Fritsche et al. was tested in a general population with headache by Yoon et al. [55] and Kukava et al. [56]. An analysis algorythm based on the ICHD criteria was used to diagnose different types of headaches, but the algorythm details were not provided [54]. The questionnaire proved to be more useful at detecting a migraine and tension type headache and it overdiagnosed patients with CH [54].

### 4.3. Images Depicting Headache Pain

Pain experience involves a complex relationship between sensory and emotional factors [57]. The diagnosis process is usually focused on the sensory experience of pain while the emotional factors are neglected [58]. Images depicting headache pain are a more complex representation of the pain experience as they also capture the affective aspect of pain [59]. This study showed that images with a higher intensity (image ‘e’ and image ‘d’) could differentiate between CH and migraine but the performance was moderate (sensitivity 70.3%, specificity 73%). Images are more precise in detecting females than males with CH. This could be due to several biases related to pain reporting as mentioned above [45,46]. Overall, the images depicting headache pain have a low influence in detecting a cluster headache. Although the intensity of pain captured via visual analogue scale and the intensity from mild to excruciating are more accurate tests than the images in detecting headache severity, they have a moderate specificity in detecting CH. This could imply that the intensity of pain is not a good differentiator between the two conditions. Other images with different artistic characteristics might have a higher performance in distinguishing between CH and migraine. Additionally, future research should capture the emotional responses evoked by images depicting headache pain in order to better understand their role in the diagnosis of primary headaches. This study is the first step in the development and validation of a screening tool for CH. As this tool was tested in a headache centre on patients who already received a diagnosis, it requires evaluation in other clinical settings, including primary care, before a diagnosis is made. A shorter screening tool should be validated, including items that best differentiated between CH and migraine according to this study.

### 4.4. Strengths and Limitations

The strength of our study consists in a large sample size and the inclusion of a control group of migraine patients, the most common misdiagnosis of CH [38]. This study opens a new field of research as it is the first study of its kind to test the usefulness of images depicting headache pain in the detection of CH, although the images in this study do not clearly differentiate between CH and migraine. The present study was subjected to limitations. The patients were recruited from a headache centre after they received a diagnosis of CH or migraine, which may have resulted in a selection bias of the enrolled patients. This study lays the foundation for the validation of the screening tool, which requires further evaluation in population-based studies. A single centre study is another limitation and it should be reproduced in other clinical settings. This study may have been subjected to recall bias, a limitation of all questionnaire studies. Our study was based on the ICHD-3b criteria, which included the recently deleted symptoms (fullness in the ear, facial and forehead flushing) from the ICHD-3 criteria and extending the maximum remission periods of chronic CH to up to three months. Although there was no difference in reporting ipsilateral ear fullness and facial flushing between patients who received a diagnosis of CH and patients who did not [60], the screening tool needs to be evaluated with the current ICHD-3 criteria.

## 5. Conclusions

This is the first study of its kind to develop a screening tool with images depicting headache pain to aid the diagnosis of CH. The overall tool score could be a good instrument in the screening of CH. Images depicting headache pain do not clearly discriminate between CH and migraine. Our study also provides valuable insight into what problems clinicians may be facing in real life to explain the delay in diagnosis.

## Figures and Tables

**Table 1:** Variables measured with the 12-item screening tool.

Question	Variable	Coded Test Scores
1. Please choose one image that best illustrates the most severe headache you have experienced	Image preference	f = 1 (least severe); a = 2; c = 3; b = 4; e = 5; d = 6 (most severe)
2. Please mark with an X the intensity of your pain on the scale below	Pain scale	Scores from zero to ten (0 = No pain; 5 = Moderate pain; 10 = Worst possible pain)
3. Please choose only one option from the following list that describes your headaches	Intensity	Mild = 1; Moderate = 2; Severe = 3; Very Severe = 4; Excruciating = 5
4. Please choose only one option from the following list that describes your headaches	Nature of pain	Throbbing/Other = 1; Pressure = 2; Stabbing/Burning = 3
5. Please choose only one option from the following list that describes your headaches	Description of pain	Red hot poker in the eye = 1; Pounding heart in the head/Other = 0
6. Do you feel restless during the headache attack?	Restlessness	No = 0; Yes = 1
7. Is the pain ‘excruciating agony’?	Excruciating agony	No = 0; Yes = 1
8. Does the pain wake you up from sleep the same time each night/ or attack comes at a specific time of the day?	Headache at specific times	No = 0; Yes = 1
9. Is the pain strictly on one side?	Strictly unilateral pain	No = 0; Yes = 1
10. Ipsilateral cranial autonomic symptoms (e.g., red watery eyes and/or runny nose?)	Ipsilateral cranial autonomic symptoms	No = 0; Yes = 1
11. How long does the most severe pain last for with medication?	Treated attack duration	> 3 h = 0; ≤ 3 h = 1;
12. How long does the most severe pain last for without medication?	Untreated attack duration	> 3 h = 0; ≤ 3 h = 1;

**Table 2:** Computation of the total score.

Variable		Minimum	Maximum
1	Image preference	1	6
2	Pain scale	0	10
3	Intensity	1	5
4	Nature of pain	1	3
5	Description of pain	0	1
6	Restlessness	0	1
7	Excruciating agony	0	1
8	Headache at specific times	0	1
9	Strictly unilateral pain	0	1
10	Ipsilateral cranial autonomic symptoms	0	1
11	Treated attack duration ≤ 3 h	0	1
12	Untreated attack duration ≤ 3 h	0	1
**Total Score**		**3**	**32**

**Table 3:** Frequency distribution of the test scores classified by diagnosis.

Test Item	Category	% within Diagnosis	
		Case Group	Control Group
		CH (*n* = 81)	Migraine (*n* = 215)
Image preference	a	4.7	18.9
	b	10.8	21.5
	c	3.9	23.3
	d	**61.9**	**18.0**
	e	8.3	7.4
	f	10.3	14.3
Pain scale	1	0.0	0.0
	2	0.0	0.0
	3	0.0	1.6
	4	0.0	2.0
	5	1.1	3.1
	6	0.0	5.4
	7	3.3	15.8
	8	3.6	33.8
	9	28.0	20.2
	10	**63.9**	**19.9**
Intensity of pain	Mild	0.0	1.1
	Moderate	0.0	13.8
	Severe	7.8	35.0
	Very severe	8.6	33.6
	Excruciating	**83.6**	**16.4**
Nature of pain	Throbbing/ Other	18.0	40.0
	Pressure	14.1	33.2
	Stabbing/ Burning	67.8	26.7
Treated attack duration	≤ 3 h	100.0	24.3
	> 3 h	0.0	75.7
Untreated attack duration	≤ 3 h	100.0	0.0
	> 3 h	0.0	100.0
Description of pain	Pounding heart/other	30.8	84.4
	Red hot poker in the eye	**69.2**	**15.6**
Restlessness	No	10	56.7
	Yes	**90.0**	**43.2**
Excruciating agony	No	10	56.7
	Yes	**90.0**	**43.2**
Attacks at specific times	No	39.1	80.6
	Yes	**60.8**	**19.4**
Strictly unilateral pain	No	13.3	55.5
	Yes	**86.6**	**44.5**
Ipsilateral cranial autonomic symptoms	No	3.6	57.8
	Yes	**96.4**	**42.2**

Key findings are highlighted in bold.

**Table 4:** Sensitivity and specificity statistics for items with dichotomous responses.

Test item	Sensitivity% (CI)	Specificity% (CI)	PPV% (CI)	NPV% (CI)	FPR% (CI)	FNR% (CI)
Description of pain	68 (58;78)	84 (79;89)	62 (52;72)	87 (83;92)	16 (11;21)	32 (22;38)
Presence of restlessness	90 (84;97)	56 (50;63)	44 (36;51)	94 (90;98)	44 (37;50)	10 (3;15)
Excruciating agony	93 (87;98)	33 (27;40)	34 (28;41)	92 (86;98)	67 (60;73)	7 (2;13)
Attacks at specific times	60 (50;71)	81 (76;86)	54 (44;65)	84 (80;89)	19 (14;24)	40 (29;46)
Strictly unilateral pain	86 (79;94)	56 (49;62)	42 (35;50)	92 (87;96)	44 (38;51)	14 (6;19)
Ipsilateral cranial autonomic symptoms	96 (92;100)	57 (50;63)	46 (38;53)	98 (95;100)	43 (37;50)	4 (0.0;7)
Treated attack duration ≤ 3 h	100 (100;100)	77 (71;82)	62 (54;70)	100 (100;100)	23 (18;29)	0 (0.0;0.0)
Untreated attack duration ≤ 3 h	100 (100;100)	100 (100;100)	100 (100;100)	100 (100;100)	0 (0.0;0.0)	0 (0.0;0.0)

PPV: positive predictive value; NPV: negative predictive value; FPR: false positive rate; FNR: false negative rate; CI: 95% confidence interval.

**Table 5:** Cut-off points on the ROC curves for whole data set.

Test Item	Cut-off	Sensitivity (%)	Specificity (%)
Nature of pain	3 (Stabbing/Burning)	67.9	73.0
Image preference	5 (Image ‘e’)	70.3	73.0
Intensity of pain	5 (Excruciating)	82.7	82.7
Pain scale	9 (out of 10)	83.9	67.9
**Total score**	**25 (out of 32)**	**86.4**	**92.0**
**Total score (no images)**	**20.5 (out of 32)**	**92.6**	**93.9**

**Table 6:** ROC cut-off points for the male and female groups.

Statistics According to Gender		Nature of Pain	Image Preference	Intensity of Pain	Pain Scale	Total Score	Total Score (without Images)
**Cut off**	Male	3	5	5	9	23.6/32	20/32
	Female	3	6	5	9	25.0/32	20.5/32
**Sensitivity** **(%)**	Male	60.7	66.6	80.3	84.3	90.1	94.1
	Female	80.0	73.3	86.6	83.3	90.0	93.3
**Specificity** **(%)**	Male	68.5	77.1	91.4	77.1	94.2	94.2
	Female	73.8	79.4	81.1	66.1	91.6	93.3

Nature of pain 3 = Stabbing/burning; Image preference 5 = Image ‘e’; Image preference = Image ‘d’; Intensity of pain 5 = Excruciating; Pain scale 9 (out of 10).

## Data Availability

The datasets used and/or analysed during this study are available from the corresponding author upon request.

## Supplementary Materials

The following are available online at https://www.mdpi.com/2076-3425/10/2/77/s1, File: The screening tool for CH.
