# Supplementary material for: Development and Evaluation of a Screening Tool to Aid the Diagnosis of Cluster Headache
Source: Brain Sci. 2020 Feb 1;10(2):77. doi: 10.3390/brainsci10020077 (PMC7071485; doi:10.3390/brainsci10020077)

**Supplementary Material: The screening tool for CH**

**Male / Female Cluster Headache**

**Age__ Episodic / Chronic**


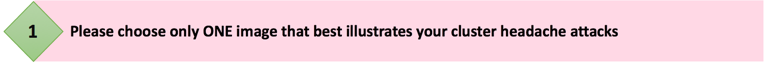


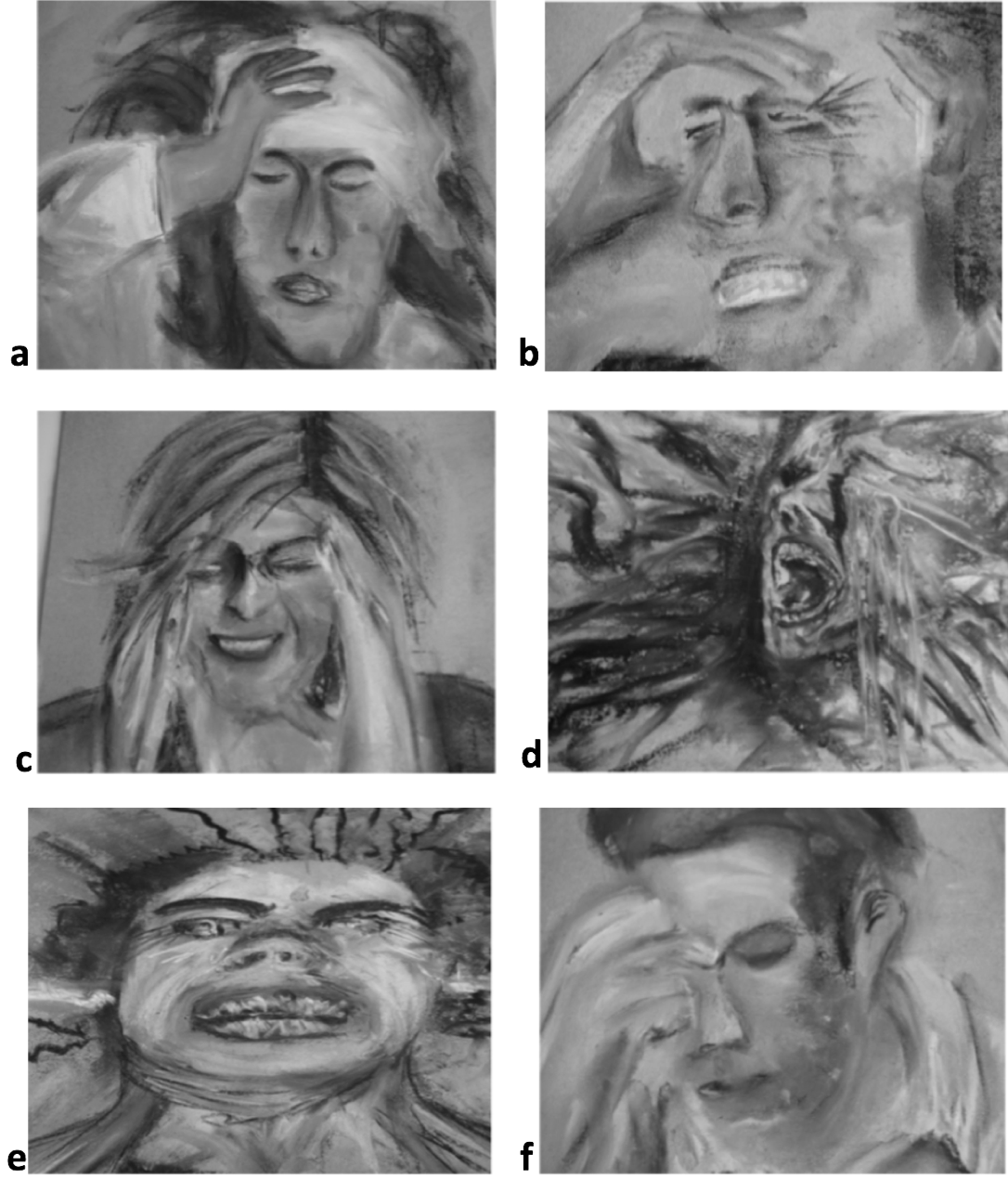


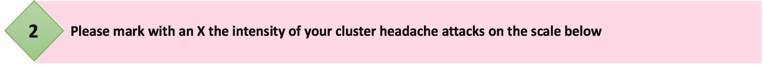


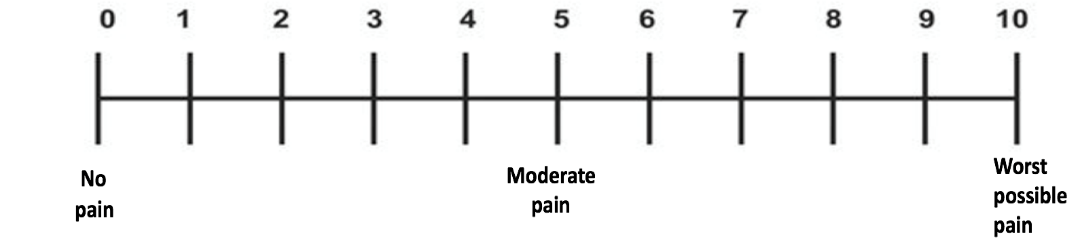


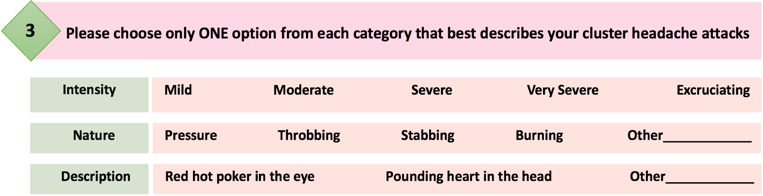


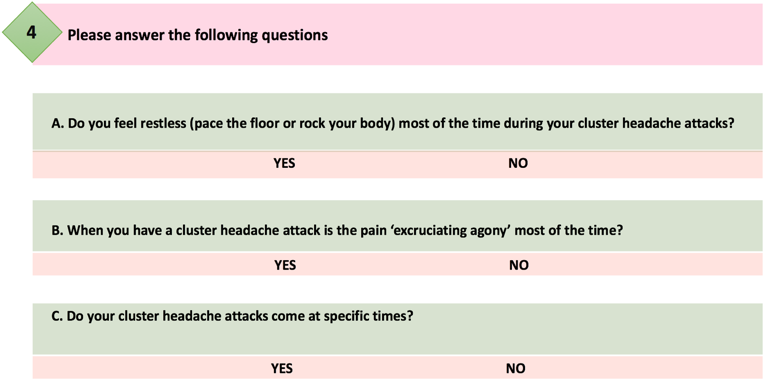


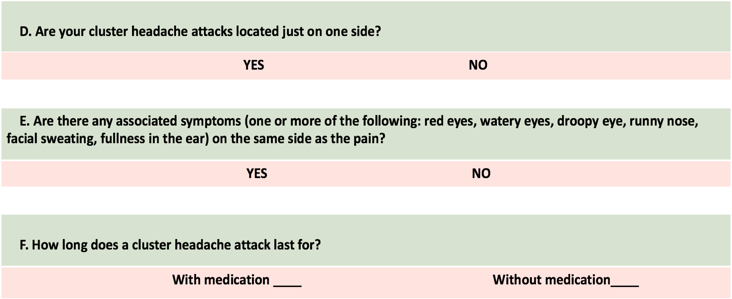

Supplement: Supplementary file 1 [file brainsci-10-00077-s001.docx]
